# The risk posed by Xanthomonas wilt disease of banana: Mapping of disease hotspots, fronts and vulnerable landscapes

**DOI:** 10.1371/journal.pone.0213691

**Published:** 2019-04-02

**Authors:** Walter Ocimati, Hein Bouwmeester, Jeroen C. J. Groot, Pablo Tittonell, David Brown, Guy Blomme

**Affiliations:** 1 Bioversity International, Kampala, Uganda; 2 Farming Systems Ecology, Wageningen University & Research, Wageningen, The Netherlands; 3 GeoSpace, The Netherlands; 4 Agroecology, Environment and Systems Group, Instituto de Investigaciones Forestales y Agropecuarias de Bariloche (IFAB), INTA-CONICET, San Carlos de Bariloche, Río Negro, Argentina; 5 Groningen Institute of Evolutionary Life Sciences, Groningen University, Groningen, The Netherlands; 6 Bioversity International, Turrialba, Cartago, Costa Rica; 7 Bioversity International, Addis Ababa, Ethiopia; Bhabha Atomic Research Centre, INDIA

## Abstract

Banana production landscapes in the African Great Lakes Region (AGLR) have been under immense pressure from Xanthomonas wilt (XW) disease over the past two decades. XW, first reported on banana in central Uganda and eastern DR Congo in 2001, has since spread to the entire AGLR. XW is currently spreading westwards from hot spots in eastern DR Congo highlands, putting the plantain (*Musa* AAB genome) belt of central and west Africa at risk. In-depth understanding of the key variables responsible for disease spread, current hotspots, and vulnerable landscapes is crucial for disease early warning and management. We mapped aggregated disease distribution and hotspots in the AGLR and identified vulnerable landscapes across African banana production zones. Available data on disease prevalence collected over 11 years was regressed against environmental and expert developed covariates to develop the AGLR XW hotspots map. For the Africa-wide risk map, precipitation, distance to hotspots, degree of trade in fresh banana products, production zone interconnectedness and banana genotype composition were used as covariates. In the AGLR, XW was mainly correlated to precipitation and disease/banana management. Altitude and temperature had unexpectedly low effects, possibly due to an overriding impact of tool-mediated spread which is part of the management covariate. In the AGLR, the eastern part of DR Congo was a large hotspot with highest vulnerability. Apart from endemic zones in the AGLR and Ethiopia, northern Mozambique was perceived as a moderate risk zone mainly due to the predominance of ‘Bluggoe’ (*Musa* ABB type) which is highly susceptible to insect-vectored transmission. Presence of XW hotspots (e.g. eastern DR Congo) and vulnerable areas with low (e.g. north-western Tanzania) or no disease (e.g. Congo basin, western DR Congo and northern Mozambique) pressure suggest key areas where proactive measures e.g. quarantines and information sharing on XW diagnosis, epidemiology, and control could be beneficial.

## Introduction

More than one third of Africa’s banana (*Musa* spp.) production, or nearly 11% of world production, comes from the African Great Lakes region (AGLR), i.e., Burundi, Democratic Republic of Congo (DR Congo), Kenya, Rwanda, Tanzania, and Uganda [1], which is a centre of diversity of East African highland bananas and plantains [2, 3]. Banana provides 30–60% of food energy needs for over 70 million people in this region [4, 5] and contributes to incomes of farm households and businessmen along the value chain of the crop [6, 7]. Since two decades, banana production landscapes in the AGLR have been subjected to immense pressure from pests and diseases on top of several abiotic constraints. The outbreak of Xanthomonas wilt disease of banana (XW) has drawn the greatest attention due its rapid rate of spread and severe impact on production. The disease was first observed on banana in 1974 in Ethiopia [8]. In the AGLR, it was first observed in 2001 in central Uganda [9] and eastern DR Congo [10], and has over a period of a decade spread to the whole AGLR [11–14]. Within the affected countries, the disease has spread to new zones at rates dependent on the agroecological conditions and the characteristics of the production systems. High spread rates have been reported in lower altitude areas (<1500 m) of central Uganda [15] with slower rates reported at high elevations (>1500 m) of eastern DR Congo [10]. The disease is currently spreading westwards from the current hot spots in eastern DR Congo, towards the Congo basin, putting the plantain belt of central and west Africa at risk. XW disease results in severe yield losses reaching as high as 100% if control is delayed, severely compromising food and income security of households and communities [16–18]. Potential economic losses between US$ 200 and 295 million a year due to delayed intervention have been estimated for Uganda [16, 19]. In Tanzania and Rwanda, a 35% drop in sales and doubling of prices due to XW were reported [20]. In Uganda, [1] reports 50% less production for 2014, compared to 2002 while area under banana declined by 39%.

Over the past 15 years, various research and extension efforts have been put in place to manage and contain the disease. For example, several XW epidemiology studies have been conducted and control strategies fine-tuned [21–30] farmers sensitized and trained; and bylaws and task forces formed to foster control [17, 31, 32]. No disease quarantines have been set up in the region to contain the disease. If attempted, low success rates are anticipated due to lack of efficient detection tools at border points, porous nature of borders and common ethnicities at borders with some households being separated by the borders [17]. Once established, landscape-wide XW control is difficult and total eradication is impossible [33]. Ocimati et al. [25] reported long incubation periods of up to 24 months and latent infections. Though helpful for reducing disease incidence and recovery of yields, the current measures have been reactive and are not adequate for containing the disease, especially not from spreading to new locations.

Knowledge of the vulnerable landscapes and disease fronts could prevent or minimize negative effects due to the disease. Here we aim to map XW incidence and identify the XW disease fronts and the vulnerable landscapes across Africa. Making XW spatially explicit can guide the design of interventions for disease management and containment. Maps will be important for surveillance; risk assessment; priority setting and resource allocation; and strategizing for disease management and containment. Mapping will also allow identification of vulnerable sites for a more pro-active disease prevention strategy, rather than the commonly applied reactive strategy. Our objectives were (i) to map the spatial spread of XW disease and the vulnerable landscapes of the AGLR, collating available disease incidence survey data and environmental and social co-variates from the various countries in the region, and (ii) to develop a first, coarse XW disease risk map for the rest of Africa. Deliberating on these XW spatial risk maps with stakeholders is anticipated to pro-actively guide decisions and strategies for XW prevention and management at landscape and regional level. This study will build on existing XW mapping exercises in the region [12, 15, 34–36] that have often been region- or country-specific.

## Materials and methods

### Study area

**Fig 1:**
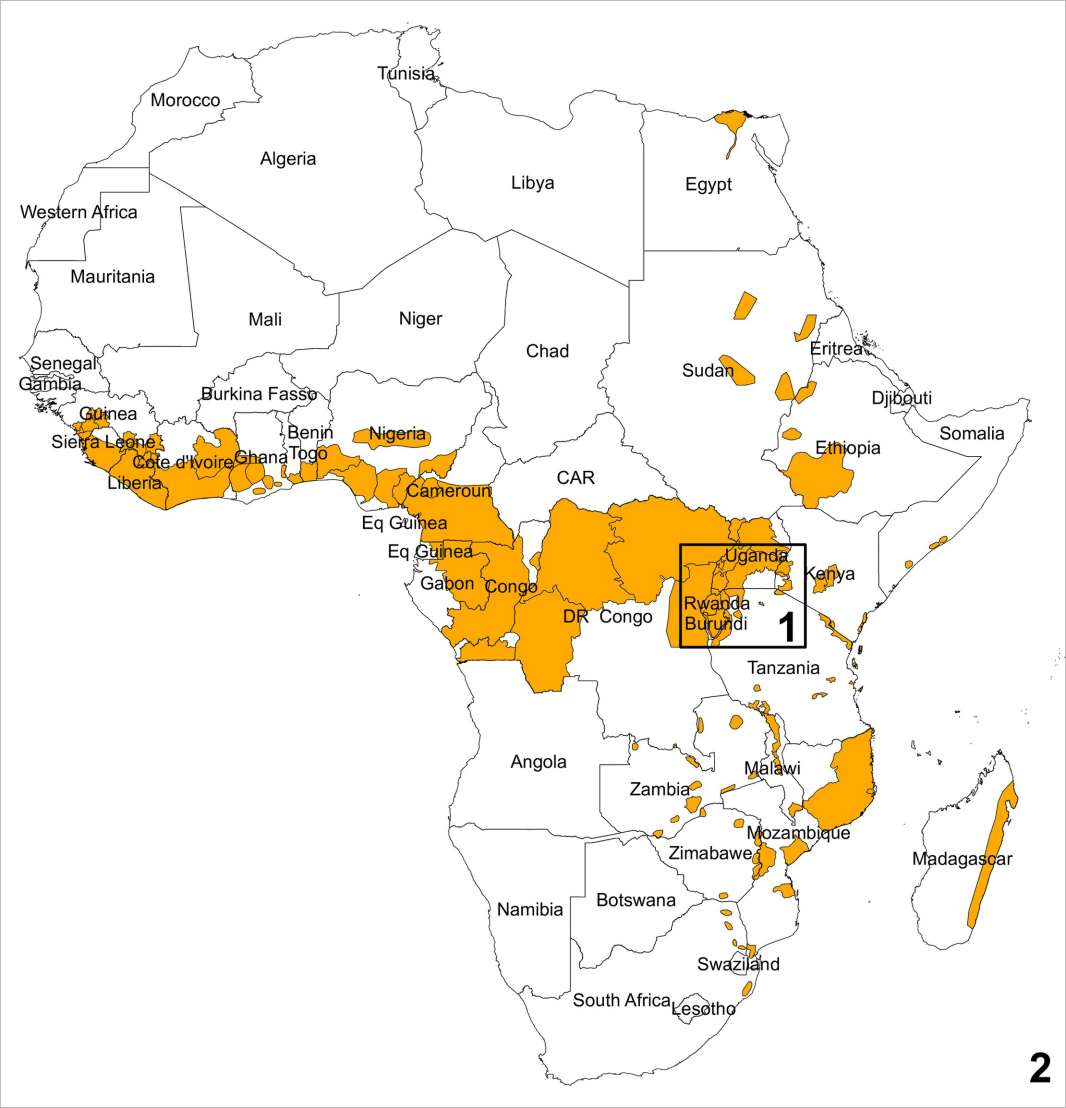
Study areas: 1) African Great Lakes region and 2) entire Africa. Shaded are the main banana growing areas (Source: www.crop-mapper.org).

The study area (Fig 1) consists of two geographical zones. The first zone, is the African Great Lakes region (AGLR) and measures about 1000 x 1000 km^2^. The area includes Rwanda, Burundi, the main banana growing areas in western Kenya, Uganda, northern Tanzania and the eastern DR Congo. The East African highlands are part of the African Rift System with Lake Victoria as the central basin. The area has a diverse agroecology, resulting from a large variation in altitude (550–4600 m above sea level; CGIAR-CSI, 2008), mean annual rainfall (500–2300 mm; [37]) and mean annual temperature (from 3 to 26 ^o^C; [37]). A large part of the area is very suitable for growing crops such as banana and plantains, maize and cassava because of fertile soils and high rainfall. Agriculture in the region can predominantly be characterized as subsistence farming with complex mixed cropping. This zone is dominated by the east African highland bananas. Plantains (AAB genome) can be found in high abundance in parts of eastern DR Congo while the ABB types can be found localized in patches across this zone, with higher concentrations in central Uganda. All surveys were carried out in this zone.

The second (much larger) zone covers all the banana growing zones in Africa, including the AGLR. The area covers 28 tropical or subtropical countries in Africa with a very diverse agroecology resulting from its sheer size and the African Rift System that cuts it in two (Fig 1). It borders two oceans, has several mountain ranges, river systems/basins and extensive forested areas. The areas in the west and central Africa are dominated by plantains while the east and central African highlands (AGLR) are dominated by the east African highland banana (AAA genome). Other AAA genomes (e.g. Cavendish types) are prevalent in the central, east and southern parts of Africa. Both study areas are delimited using administrative boundaries (Global Administrative Areas vs. 2.8; www.gadm.org).

### Methodology for zone 1 (i.e. AGLR)

#### Data collection

**Fig 2:**
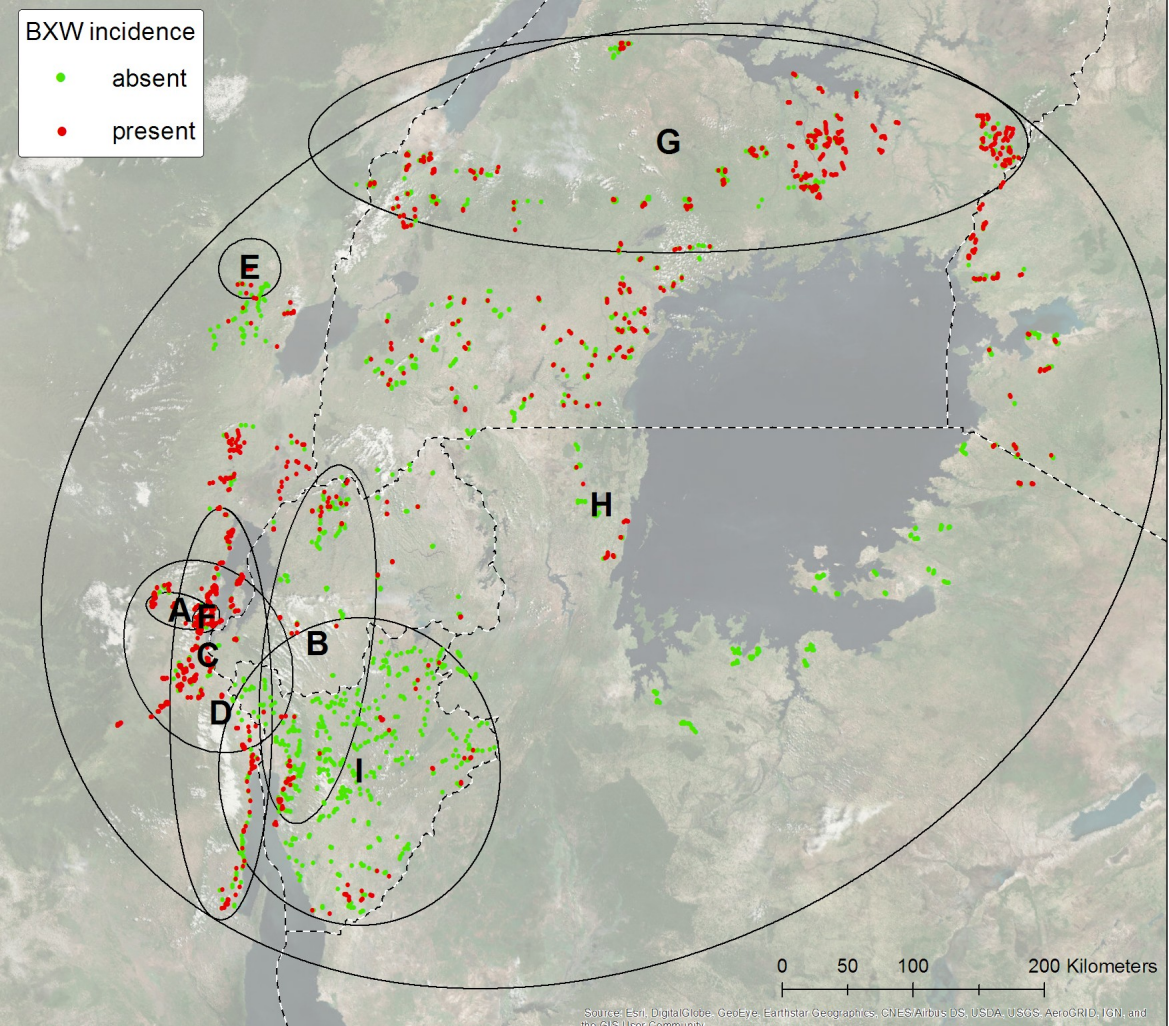
Incidence of XW for all nine surveys indicated with letters A to H. An ellipse/circle shows the geographic extent of a survey.

Survey datasets: The data set for mapping the AGLR was collated from nine ground-based surveys conducted between 2005 and 2016 across the zone 1 (Figs 1 and 2) and comprised of a total of 4,760 farms. An ellipse is added for each survey to show its geographic extent (Fig 2). The smallest extent is that of survey E, where all samples lie within a 3 km distance. The largest one is survey H, where the samples lie within 1000 km distance. Only survey H covers the entire area of interest. XW incidence/ distribution was recorded through farmer interviews and diagnosis of banana farms/fields. The coordinates of the sample locations were recorded with a handheld GPS with an error margin of about 10 m. The surveys measured XW as binominal (present or absent) or categorical values (percentage incidence of infected plants in a farm (i.e. 0 to 100%)). For uniformity, all measurements were transformed to binominal values (1 = present or 0 = absent).

**Fig 3:**
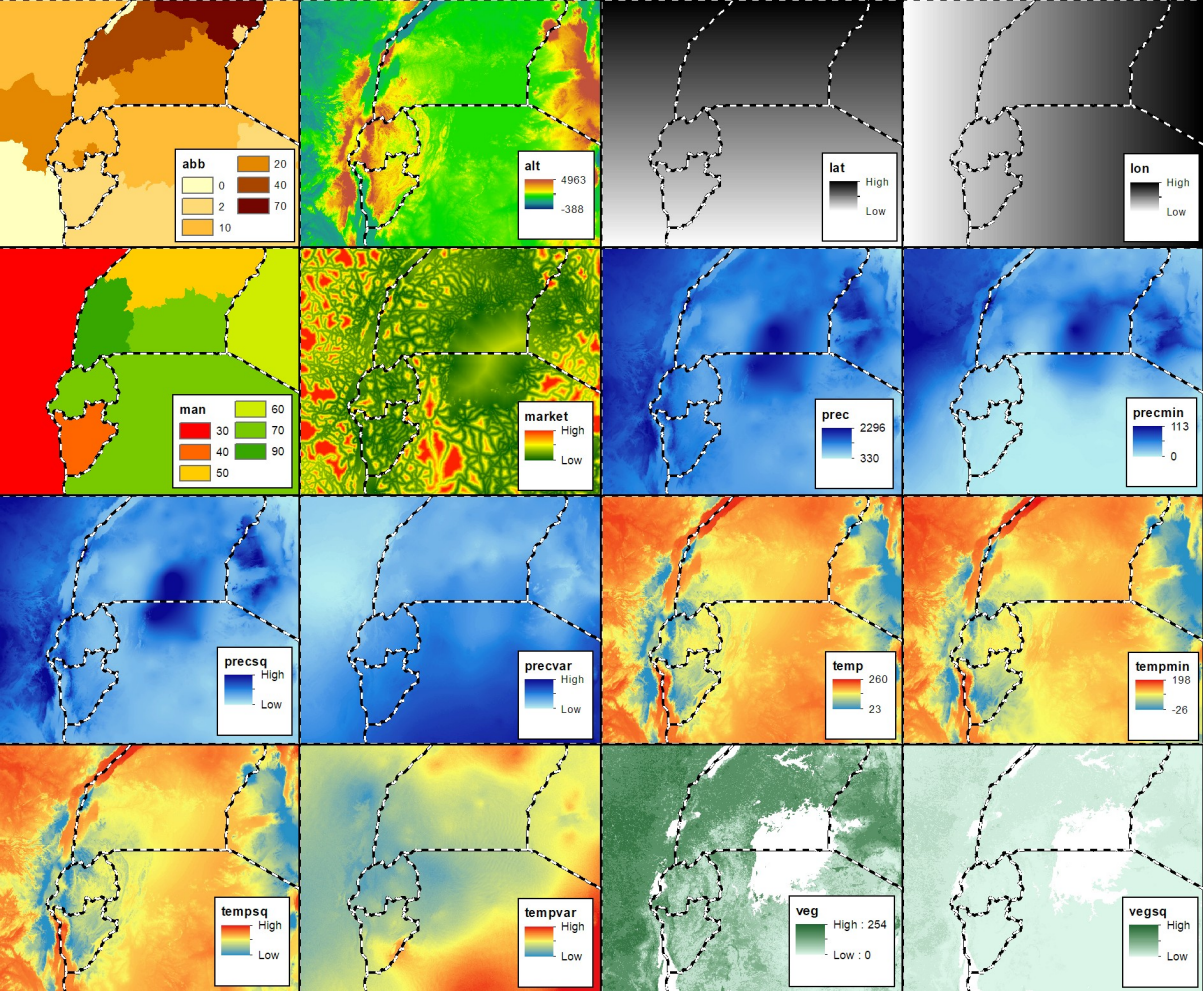
Covariate maps that served as input for the regression model for developing the spatial map of Xanthomonas wilt disease in the African Great Lakes Region.

Dataset of covariates: Most of the area of interest for the AGLR risk mapping is devoid of samples. Environmental variables (e.g. altitude, rainfall, temperature) and banana cultivar composition have a relationship with XW disease [17, 21, 35, 36, 38]. Precipitation, temperature and altitude influence insect vector activity and thus the incidence and severity of XW disease [21, 39, 40]. The ABB banana types are particularly prone to insect-mediated infections due to their non-persistent neuter flowers [18], as such depending on their concentrations and farm management practices, landscapes containing them tend to be more prone to XW infection. Environmental characteristics/banana types can thus help to predict the incidence of XW or the risk of infection at the unobserved locations. The environmental variables (e.g. topography, vegetation, temperature, precipitation) and major markets were obtained from publicly available predictor maps. Data on the distribution and abundance of ABB types in the AGLR was obtained through literature review and expert knowledge. The level and organization of extension services and thus disease management also plays a key role in the spread, incidence and severity of the disease. To capture this, through expert judgement, management was incorporated as an additional covariate using a scale varying between 0 and 1, ‘0’ denoting no efforts to manage XW disease and ‘1’ strong research, extension and management efforts to control XW. Table 1 lists all environmental variables that have been used for mapping the AGLR and Fig 3 illustrates them as maps.

**Table 1:** Covariates used for the regression analysis and for making the infection risk map of the African Great Lakes Region.

	Var.	Description	Resolution	Source
1	lon	Longitude	30 arc s	-
2	Lat	Latitude	30 arc s	-
3	Alt	Altitude above sea level (m)	30 arc s	http://srtm.csi.cgiar.org [41].
4	prec	Annual precipitation (mm)	30 arc s	WorldClim V1 [37]
5	precsq	-	30 arc s	-
6	precmin	Precipitation driest month (mm)	30 arc s	WorldClim V1 [37]
7	precvar	Precipitation seasonality (-)	30 arc s	WorldClim V1 [37]
8	temp	Mean annual temperature (°C)	30 arc s	WorldClim V1 [37]
9	tempsq	-	30 arc s	-
10	tempmin	Mean temperature coldest month (°C)	30 arc s	WorldClim V1 [37]
11	tempvar	Temperature seasonality	30 arc s	WorldClim V1 [37]
12	market	Market access (hr)	30 arc s	www.forobs.jrc.ec.europa.eu
13	veg	Vegetation cover	30 arc s	ESA CCI landcover map V1
14	Vegsq	-	30 arc s	-
15	Man	Management Factor (-)	30 arc s	Expert knowledge
16	abb	Distribution of ABB banana types	30 arc s	Expert knowledge

#### Indicator regression kriging

To develop the XW disease map for the AGLR, the indicator regression kriging method was used. Indicator regression kriging is a geostatistical interpolation method that spatially interpolates a response variable, making use of point observations of the target variable and auxiliary data [42, 43]. Bouwmeester et al. [35], used the method to map XW in the East African highlands. In this study, the same technique was applied, but multiple merged survey datasets were used, instead of just one, and hence the spread of XW was predicted for a larger area. Bouwmeester et al. [35] used data of 2006/7 only, and since then the disease has spread to other areas in the region. The methodology involves two stages. In the first stage, the binary survey variables are regressed to auxiliary environmental (e.g., terrain, climate, land cover) and social (e.g. management) covariates. In the second stage, the regression residuals are interpolated using simple kriging and added to the regression map to further improve the spatial prediction of XW incidence across the entire region.

Regression analysis: Regression models predict at the unobserved locations using the relationship between the observed locations and the environmental auxiliary covariates. We used 16 auxiliary covariates (Table 1) that were thought to have a plausible and significant relationship with XW or its vectors and for which spatially exhaustive maps in the public domain or literature and knowledge were available. An overlay of the survey locations and covariates resulted in a database with XW presence and 16 covariates. This database served as input for fitting the regression models. We applied logistic regression because the dependent variable (XW presence) is binary, either 1 for present or 0 for absent. The theory and practical application of logistic regression is well explained by [44] and by Bouwmeester et al. [35] and therefore only briefly described it in this article. The general logistic regression model was built in four steps:

1. All 16 covariates (Table 1) were entered into a univariate logistic regression model. Covariates with a significance level less than 0.25 were included in further analysis because these may be significant in two-way interactions.
2. The 16 covariates (Table 1) were then entered into a multivariate logistic regression model. With stepwise regression, the covariates that were not significant at the p = 0.05 level were removed one at a time.
3. Two-way-interactions between covariates were included to check for combined effects that improve the likelihood ratio. Initially, all possible covariate interactions were examined. All interactions with a p-value less than 0.05 are deemed significant and included in the model, again using a stepwise approach.
4. The goodness-of-fit of the final model was assessed in terms of deviance and compared to the null model (i.e., the model without covariates) using the likelihood ratio test.

Finally, the derived logistic regression model was used on the covariate maps, and yields a regression prediction map for zone 1 of the study area.

The R Statistical software [45] and the Raster package [46] were used for the regression analysis.

Spatial interpolation: In most cases, the regression model will only describe part of the variation in the response variables. Through regression kriging, the regression residuals were interpolated with kriging and used to correct the estimate of the regression model. Kriging predicts at unobserved locations by taking a weighted average of the surrounding observations, where the kriging weights depend on the spatial autocorrelation between the variable at the prediction and observation locations. The spatial autocorrelation is characterized by the semivariogram that plots the semivariance, i.e., a measure of the degree of variation, as a function of geographical distance [42]. In regression kriging, the residuals from the regression analysis are used instead of the observations directly. We estimated the semivariogram model parameters based on visual interpretation of the semivariance plot. Next, simple kriging of the regression residuals was applied since the regression residuals have zero mean [43]. Finally, the simple kriging method yields a kriging prediction map for zone 1 of the study area. The GSTAT package in R [47] was used for this.

#### XW prediction map

To predict the XW incidence of the entire region, the regression prediction map was simply added to the kriging prediction map. Cells were set to zero if the sum of the regression- and the kriging-values was negative and cells were set to 1 if the sum exceeded 1.

### Methodology for zone 2 (banana growing areas in Africa)

For the development of XW infection risk map at the Africa-wide level, we cannot directly rely on the current surveys because these cover only the AGLR. Therefore, variation in environmental covariates is beyond the range of the samples, and the calculated relationships for study area 1 will not hold. Also, XW is currently not present in the lowland plantain growing zones of west and central Africa and not in the Cavendish (AAA genome) growing zones in eastern and southern Africa. Management as a covariate would also not work for the Africa wide context due to the absence of the disease.

**Fig 4:**
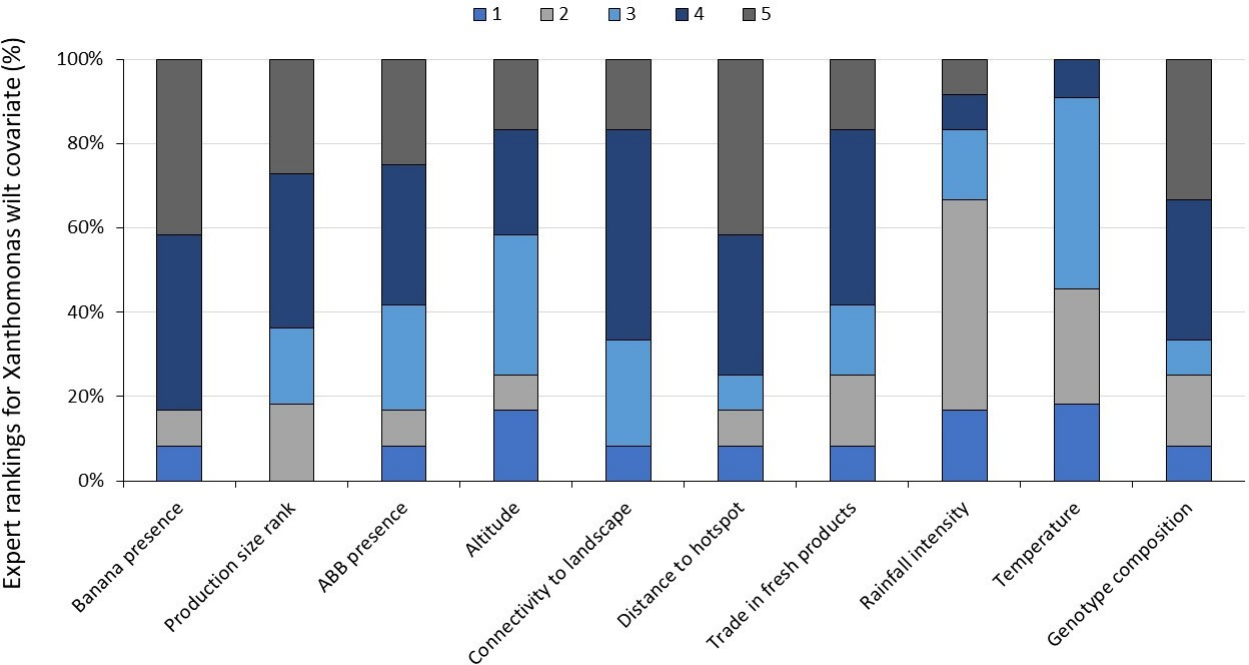
The percentage of experts giving different ranks for 10 potential covariates influencing risk of Xanthomonas wilt disease spread and severity. Covariates were ranked at a scale of 1 to 5, “1” denoting that a covariate does not influence XW disease while “5” has a very important influence on XW disease. Fourteen experts participated in ranking of the covariates.

To overcome this challenge, a prediction of infection risk was estimated by calculating a background risk level to all banana growing zones in Africa based on the relationship between XW disease and selected covariates (environmental and expert generated). This background risk was then corrected based on expert knowledge. The expert assessment of covariates was attained through a two-step procedure. In the first step, a questionnaire was administered to 14 experts on XW epidemiology, to determine the importance of ten suggested covariates on a scale of 1–5 (Fig 4). Rank ‘1’ stands for not important while rank ‘5’ denotes very important in influencing XW disease. Table 2 lists the covariates that were finally used for the Africa wide XW risk map. Subsequently, and using the selected covariates, three experts with good understanding of XW disease epidemiology and the banana production zones, independently ranked the risk scores for each production zone/ country.

**Table 2:** The covariate data used for developing the Xanthomonas wilt of banana risk map for production zones in Africa.

Covariate	Detail	Source	*μ*	Σ	Weight
Zones	Banana production zone	www.crop-mapper.org and http://mapspam.info	(-)	(-)	(-)
Genotype	Banana genotype composition. ABB types are prone to insect vector-mediated infections predisposing landscapes to XW [12].	www.crop-mapper.org Various banana experts and authors.	1.3	0.6	+1
Connectivity	Interconnectedness to hot spots—isolated areas have a lower exposure to XW. Could be influenced by a common border, main road axis, water body or forests.	Various banana experts and authors.	0.3	0.4	+1
Trade	Trade with hotspots in fresh fruits. Spread along trade routes has been reported [27, 50].	Various banana experts and authors.	0.4	0.5	+1
Distance	Actual distance to hotspots—closer a site is to a hotspot the higher the XW risk	Various banana experts and authors.	14.0	12.8	-1
Precipitation	Annual precipitation (mm)- higher number of infections reported in the wet seasons.	WorldClim V1 [37]	1333.4	596.7	+1

Most of the banana production zones for which the XW infection risk was calculated were downloaded from the crop-mapper application (www.crop-mapper.org). The zones were modified using ArcGIS [48] because some zones were 1) overlapping, 2) were entered twice, 3) were split into small polygons or 4) were displaced. In some countries, zones were added because they were missing in the crop-mapper application despite having significant banana production [1]. The geographic location of these missing zones was based on the maps of MapSPAM V2 [49] banana production.

After editing there were 121 zones within 28 African countries. In some countries there were many zones with a maximum in Guinea of 15, whereas in other countries like Madagascar there was only one zone. To each zone relevant covariate data were added. The relevance was determined through expert assessments and by interpreting correlation coefficients between environmental data and XW samples in the East African Highlands (Table 2).

The values of the covariates had very different ranges (Table 2). To make comparisons possible all values were standardized. To calculate the infection risk of each zone, five covariates (genotype, connectivity, trade, precipitation and distance) were simply summed. Four other covariates (e.g. production zone size, production level, temperature and altitude) were also considered but not used in the end because the resulting maps suggested a non-useful relation. It was assumed that the covariates genotype, connectivity, trade and precipitation increase risk, whereas distance decreases risk. It was also assumed that all covariates have an equal weight in determining risk.

## Results

### Correlation of covariates to XW occurrence in AGLR

Pearson’s correlation between XW incidence and all covariates were significant at the p = 0.05 level, except for the two covariates market and vegetation squared (Vegsq) (Table 3). Three covariates (precipitation squared, precipitation and management) had an absolute correlation coefficient of 0.35 or higher, suggesting a higher contribution to the observed variation in XW disease occurrence. All precipitation covariates, except the variability in precipitation, were positively correlated to XW. This suggests XW incidence is higher during the wet/humid seasons. In contrast, the disease management factor was negatively correlated with the XW incidence. Thus, a higher occurrence of XW is expected in landscapes that either had no access to management information or in which disease was poorly managed.

**Table 3:** Pearson’s correlation coefficients between XW incidence and covariates used for the African Great Lakes Region map, ordered from highest positive to high negative.

Variable code	Variable name	Pearson correlation coefficient (r)	p-value
Precsq	Precipitation squared	0.41	2.2e-16
Prec	Annual precipitation (mm)	0.40	2.2e-16
Alt	Altitude above sea level (m)	0.14	2.2e-16
Precmin	Precipitation driest month (mm)	0.09	1.6e-10
Veg	Vegetation cover	0.09	6.9e-10
Abb	Distribution of ABB banana types	0.03	0.035
Market	Market access (hr)	0.02	0.142
Vegsq	Vegetation cover squared	0.02	0.112
Lat	Latitude	-0.09	2.8e-09
Tempmin	Mean temperature coldest month (°C)	-0.10	2.3e-11
Tempsq	Temperature squared	-0.14	2.2e-16
Temp	Mean annual temperature (°C)	-0.15	2.2e-16
Precvar	Precipitation seasonality (-)	-0.17	2.2e-16
Tempvar	Temperature seasonality	-0.18	2.2e-16
Lon	Longitude	-0.22	2.2e-16
Man	Management Factor (-)	-0.35	2.2e-16

### Regression analysis and spatial interpolation

**Fig 5:**
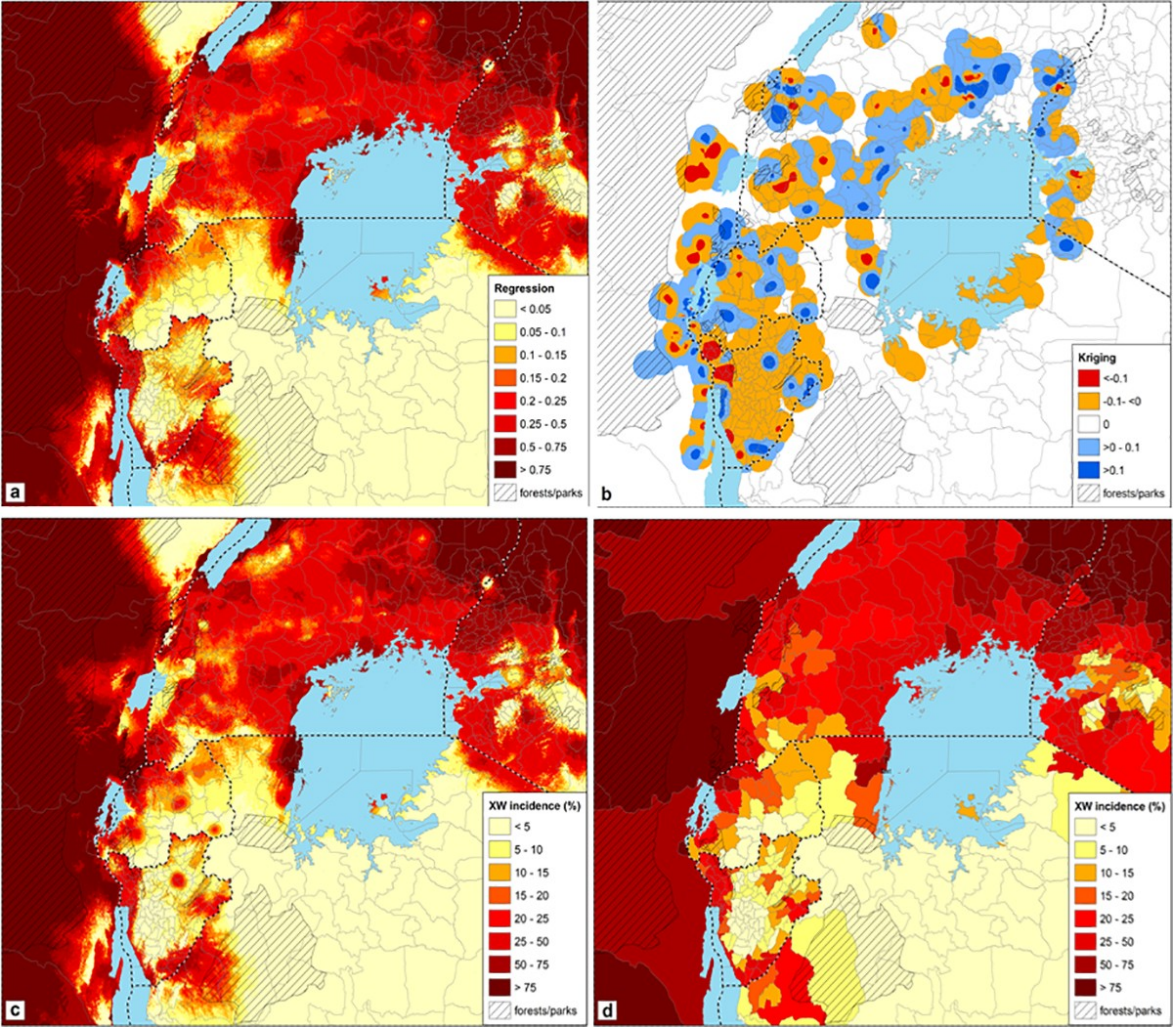
**The XW incidence in the AGLR (c) is the sum of the regression prediction map (a) and kriging prediction map (b) and is aggregated by administrative boundaries (d).** Large lakes are shaded blue. Areas deemed unsuitable for banana production, above 2500 m, forests and or national parks are denoted by black stripes/ dashed lines and correspond to masked areas.

The deviance of the regression model was 25.9% smaller than the deviance of the null model, which indicates the covariates (S1 Appendix) improved the goodness of fit of the model and explain a larger part of the variation than the null model. The covariates management (‘man’), precipitation seasonality (‘precvar’), precipitation in the driest month (‘precmin’) and precipitation (‘prec’) (S1 Appendix) had relatively high absolute values, just like the correlation values (Table 3). Similar to observations with the correlations, this suggests that the management and the precipitation related covariates had the highest contribution to the observed variation in the model, thus XW occurrence. A shift in the sign for ‘prec’ and relative importance of covariates is observed in the regression coefficients (S1 Appendix) in comparison with correlation values (cf. Table 3). These changes may be explained by the fact that the covariates are themselves correlated, which is very much the case between ‘prec’ and ‘precsq’ with a correlation efficient close to 1. In the regression model all covariates and interactions are lumped together in multivariate model, whereas the correlations are univariate. In the multivariate regression model the contribution of a specific covariate may entirely be covered by another one, as is probably the case for ‘prec’. With the final regression model a regression map was calculated (Fig 5A).

The kriging prediction map was calculated from the regression residuals. First a semivariogram was calculated (S2 Appendix). It is a near nugget variogram, meaning that there is little spatial autocorrelation between residuals. With the semivariogram a kriging prediction map was calculated (Fig 5B). The map consists of different colour shades around the samples representing relatively small positive or negative prediction values. In the blue areas the sample kriging prediction values were higher than the regression model predictions (i.e. >0) whereas in the red areas the values were lower (i.e. <0). The kriging prediction value in most parts of the study area is zero, the average value of the residuals, i.e. the kriging predictions were the same as the regression predictions.

### XW incidence map of the African Great Lakes region

The XW incidence map of the East African Great Lakes region (Fig 5C) is the sum of the regression predictions (Fig 5A) and the kriging predictions (Fig 5B). The areas with black stripes/ dashed lines correspond to masked areas, where the altitude is above 2500 m, forests or national parks and deemed unsuitable for banana production. The resulting high-resolution map had a cell size of approximately 1 km, which may be too detailed and difficult to translate into clear-cut policy decisions or recommendations. Therefore, an aggregated map showing the XW incidence per district was created by calculating the average value for all cells in a district (Fig 5D).

Both, the high resolution and aggregated XW incidence maps strongly resemble the regression map as the influence of the kriging is limited and local. The maps show that the risk XW spreads beyond the original data points (Fig 2) given the biophysical conditions and management is high. The disease has a high likelihood of being in all districts of Uganda and eastern DR Congo. The eastern part of DR Congo is a potential large hotspot with high XW occurrence. Uganda has a moderate to high XW occurrence/incidence. Clusters of potential XW hotspots were also visible in the Kagera region of north-western Tanzania, western parts of Burundi and Rwanda, southern Burundi and in western Kenya at the border with Uganda. Large portions of Burundi, Rwanda and the banana producing zones in Tanzania, have low levels of or no likelihood of appearance of XW. However, large portions of the survey regions were devoid of data (as surveys did not cover all banana production regions), though could potentially be having the disease.

### XW infection risk in tropical Africa

**Fig 6:**
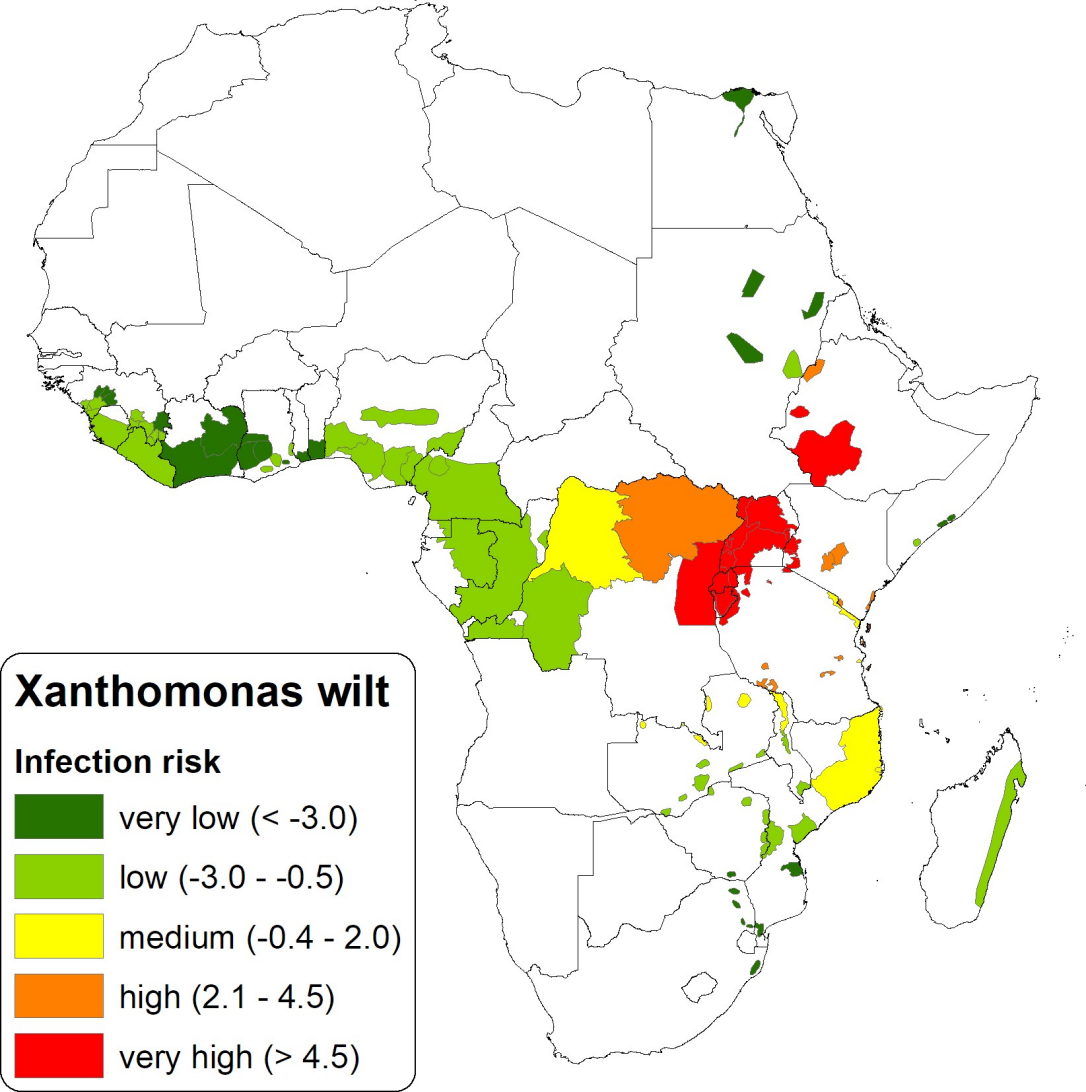
Infection risk of Xanthomonas wilt in tropical Africa developed using five expert generated covariates i.e. banana genotype, connectivity to disease hotspots, trade with an infected zone or country, precipitation and distance from a hotspot.

The XW infection risk for all banana production zones in tropical Africa derived from expert developed covariates (Fig 6) shows one zone in Tanzania, two zones in Ethiopia and the infected zones in the AGLR to have a very high risk (>4.5) due to the presence of the disease in these zones. A high-risk score of 2.1–4.5 was observed for one production zone in Mozambique, a large zone stretching from north eastern-central DR Congo, a zone in northern Ethiopia, zones in Kenya and Tanzania. Production zones in Egypt, Sudan, South Africa, Guinea, Togo, Ghana and Cote d'Ivoire had the least risk to XW infection.

**Fig 7:**
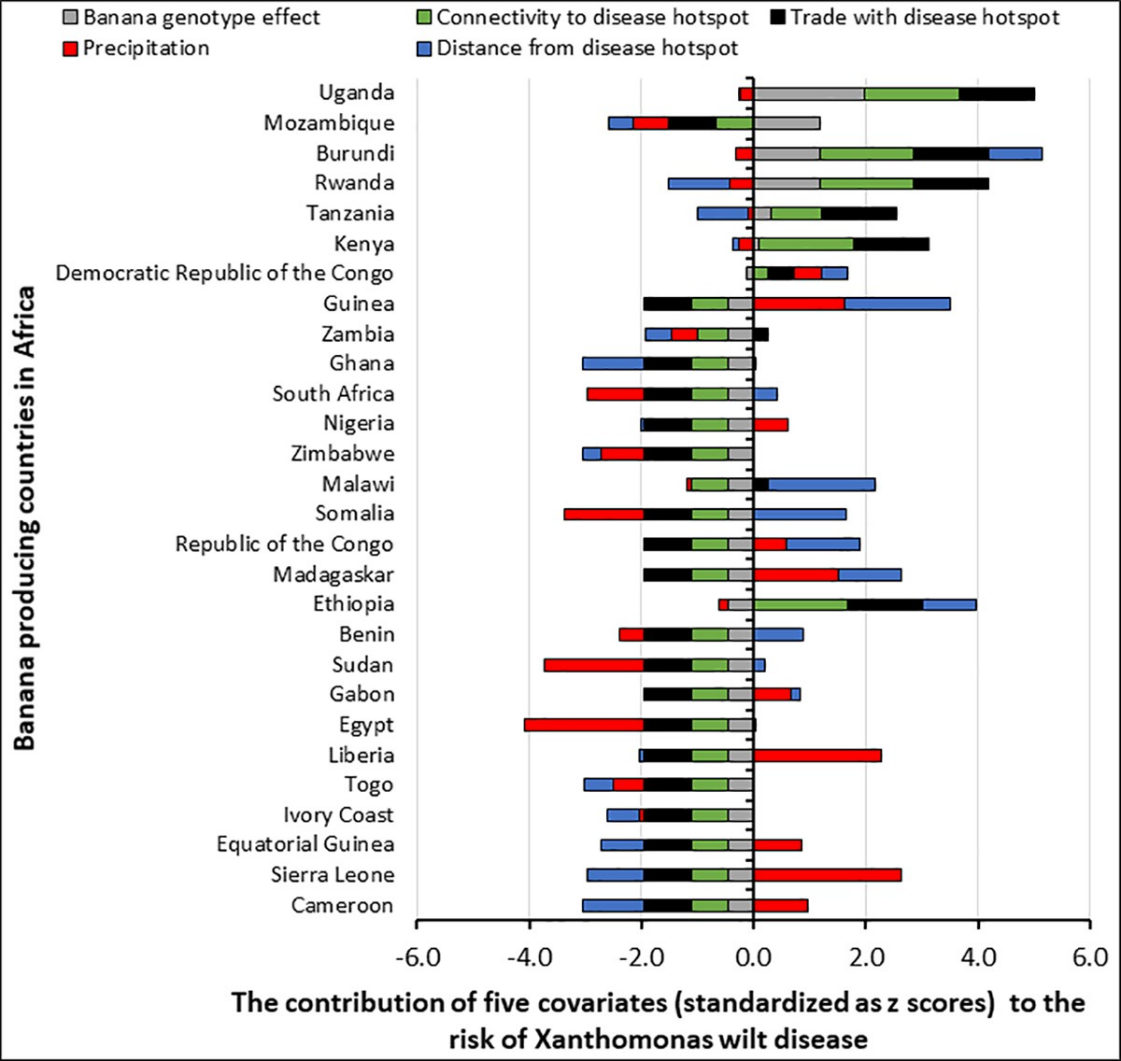
The contribution of five expert developed covariates (i.e. trade with a disease hotspot, connectivity to a disease hotspot, precipitation, distance from a disease hotspot and banana genotype composition) to the Xanthomonas wilt disease risk levels of different countries that grow banana in Africa. Covariate scores have been standardized as Z scores.

Banana genotype effects on the landscape risk of XW were strong in Mozambique and parts of the AGLR (Fig 7). Connectivity of landscapes and inter country trade also had strong contributions to the XW risk in the AGLR. Presence of the ABB-genotypes that are prone to insect-mediated infections, connectivity to a hotspot, and trade increase the risk of exposure to XW disease. Risk variability in the western part of Africa was mainly influenced by the distance from the hotspots and the level of precipitation (Fig 7). A long distance from the hot spot(s) and a low precipitation is associated with a lower risk. Risk in the northern parts of Africa were mainly influenced by the low amounts of precipitation.

## Discussion

Xanthomonas wilt disease of banana has rapidly spread across the AGLR and the plantain belt of central and west Africa is currently at risk. This study developed risk maps showing the aggregated spatial XW disease distribution and hotspots in the AGLR, and vulnerable landscapes across African banana and plantain production zones (Figs 5 and 6).

### XW occurrence and incidence in endemic zones of the African Great Lakes region

In the endemic zones of the AGLR the occurrence and incidence of the XW disease was largely explained by precipitation and management (cf. Table 3, S1 Appendix). The occurrence of XW increased with precipitation and declined with increasing level of disease/banana management.

High precipitation offers a conducive environment for both the pathogen (*Xanthomonas campestris* pv. *musacearum* (Xcm)) and the host [51]. Higher XW severity and incidence has often been reported on farms during the rainy season in contrast to the drier seasons [12, 38]. Using Maxent and regression, [36] also reported precipitation to be positively correlated to XW and to predominantly explain XW development in Tanzania. Rainfall and water availability can affect the survival, vigor, multiplication, spore production, inoculum dispersion, spore germination and penetration of a pathogen [51–54].

A humid microclimate within the crop can result in stomatal opening allowing microbes to enter the plant apoplast [54] and also modulate bacterial population and disease incidence [55, 56]. Xcm bacteria are in the group of proteobacteria, that are sensitive to desiccation [57, 58] and thus likely to be favored by high humidity in plant tissues. Results from [30] show banana plants that receive an adlib supply of water to be more susceptible than those that received a moderate water stress, and observed that banana plants tended to remain in a latent state when moisture in the soil was deficient. High humidity has been associated with suppression of R gene mediated Hypersensitive Response that involves rapid plant cell death at point of infection in some plant species [59], though not yet investigated or reported for Xcm.

Shimwela et al. [36] suggests the short distance spread of XW causing bacteria through rain splashes as the possible explanation for the higher correlation of XW infections to high rainfall or the rainy season. Infections through rain splashes would however be feasible in the presence of wounds on healthy plants and bacterial ooze on the ground and inflorescences, that would be most likely associated with farm management practices or pest damage. For example, Xcm has been reported to infect plants and cause disease if it comes in contact with fresh wounds on the roots or corms resulting from nematode or tool damage [26, 60].

Management plays a crucial role at regulating disease pressure on farms. XW is spread over both short (at field level and between close fields and farms) and long distances mainly through contaminated farm tools, insect vectors and infected planting material and occasionally through fruit/nectar feeding birds and bats [17]. To prevent spread or reduce disease inoculum and incidence, tool sterilization, early male bud removal using forked sticks (prevents insect-mediated infections), removal of infected bunches, plants and or mats are recommended. Where these practices have been applied, the disease has been contained or kept to lower levels of severity or incidence [17,18, 21, 22, 32, 33, 38]. Increased use of farm tools most often without sterilization in the rainy season during field preparation, pruning of leaves to introduce annual crops could also contribute to the higher incidence in the rainy season [12, 17, 18].

Altitude and temperature have also been reported to influence XW spread, mainly through their effect on insect vector activity. Insect vector numbers and population activity is lower at higher and cooler altitudes resulting in a lower disease occurrence and severity [39, 40]. But unexpectedly low correlation and regression coefficients between XW and temperature and altitude were obtained, possibly due to the larger impact of tool-mediated XW spread (captured in the disease management covariate–cf. Table 3, S1 Appendix). More so, the endemic AGLR sites are dominated by the east African highland banana types that are not very prone to insect-mediated XW transmission given most of them have persistent male floral bracts and flowers [12].

In the AGLR, multiple disease hotspots were observed in the entire study area (Fig 5). The eastern part of DR Congo was a large hotspot, while Tanzania had most of its production zones XW free (cf. Fig 5). The XW status in eastern DR Congo could be attributed to the lower level of control/ management efforts due to a weaker extension support system. A recent study in eastern DR Congo reported a low adoption of XW management practices, while only 32.3% of farmers had accessed some training on XW management over the past five years [61]. The distribution of this training was skewed with some regions having zero access. The eastern DR Congo also has high precipitation that has been shown in this study and studies of [35] and [36] to be correlated with high infection levels. In contrast, Rwanda had a strong extension effort, including a mandatory government driven effort to uproot swathes of plantations in disease hotspots in western Rwanda with plans to reintroduce the crop after a few years of fallow [62]. In contrast, production zones in Tanzania are distant from each other, preventing the spread of XW.

Clusters of XW hotspots were also visible in the Kagera region of Tanzania, central and eastern regions of Uganda, the western part of Burundi and in the western part of Kenya on the border with Uganda. This could be attributed to the rapid rate of spread over short distances (e.g. though contaminated farm tools, insect vectors, small ruminants, infected planting materials and rain splashes/floods).

### XW infection risk in tropical Africa

Apart from the endemic zones in the AGLR and Ethiopia, northern Mozambique was perceived to be at a very high risk mainly due to the omnipresence in backyards of ‘Bluggoe’ (*Musa* ABB type) which is highly susceptible to insect, bird and bat-vectored transmission. Ocimati et al. [63], observed a significant association between the presence/absence of the XW-susceptible ABB types with disease incidence on a farm. The ABB banana types have also been blamed for the rapid spread of XW in Uganda (period 2003 till 2006) from the initial point of infection in Luwero district in Central Uganda. Proactive preventive measures in both the southern part of Tanzania and northern part of Mozambique and Malawi will be crucial for preventing a southward spread of the disease. These could include community awareness to improve surveillance and introduction of infected planting materials or fresh products and installation of quarantine measures. Similar measures are also needed to prevent the westward spread of the disease into the Congo basin and the plantain growing belt of west Africa.

### Reflection on the methods

The surveys carried out in the AGLR were an accurate method of scouting for XW disease. However, their reach was limited by the need of a high financial investment, time constraints and limited access to some of the study locations (leading to convenience sampling). The study shows that geostatistical approaches can overcome the above challenges and use limited surveys or data points to make valid and precise predictions beyond the surveyed areas. Bouwmeester et al. [34], through cross-validation reported regression kriging to yield unbiased predictions of XW occurrence. The regression model however suffers some limitations and may not as such capture part of the variation in XW. First, not all underlying processes that cause spatial variation in XW incidence e.g., mode of transmission, distribution of susceptible host types and level of disease management are known or can be effectively represented by covariates in the model. The mode of spread of XW is complex, involving mainly farming tools, insect vector spread and planting materials. Spread through these modes can be minimized through cultural management practices. In the current study, a management covariate based on expert knowledge and available literature was incorporated to capture some of these aspects (cf. Table 1, Fig 3). The ABB *Musa* types are also known to be highly susceptible and a covariate on the distribution of ABB *Musa* types (cf. Table 1, Fig 3) was as such incorporated on the basis of expert knowledge and available literature. These covariates based on expert knowledge may suffer from errors due to failure to capture minor details e.g. variations over short distances and a lack appropriate scale but give valid predictions over large geographical scopes. For the Africa-wide risk map, a prediction of infection risk was estimated based on the relationship between XW disease and selected environmental and expert developed covariates because the variation in environmental covariates outside of the AGLR was beyond the scope of the surveys. These covariates could as such suffer from errors and or lack appropriate scale. However, this exercise gives us the first coarse XW disease risk map for the rest of Africa that can offer a platform to pro-actively make decisions and strategies for containing the disease to the currently affected zones.

## Conclusion

XW is spread across most of the AGLR. All banana landscapes in this region are vulnerable. Efforts in the region could focus on managing/reducing the disease and its damage on productivity. Landscapes with high precipitation are hotspots of XW or highly vulnerable to XW infection. Management plays a crucial role on the current XW incidence and prevalence. Improving extension services is crucial for the management of the disease in the AGLR. Extension efforts should be concentrated to such landscapes to curb or reduce the XW pandemic. Production zones in northern Mozambique and central lowland DR Congo are potential gateways for the spread of XW southwards and eastwards, respectively. Proactive measures are crucial for the prevention of the disease to these production zones. Possible actions could include the institution of quarantine measures and provision of relevant information and training on diseases diagnosis and epidemiology. The integration of expert judgement in development of covariates that are not readily available yet capture underlying processes that cause spatial variation was crucial in improving the regression outcomes.

## Supporting information

S1 Appendix Estimates and standard errors of coefficients of the logistic regression model. Interactions are indicated with the ‘:’ sign.
(PDF)

S2 Appendix The semivariogram of the regression residuals (nugget = 0.14, sill = 0.17, range = 0.2 degrees). The line depicts the variogram model, with little spatial autocorrelation until a distance of 0.2 degrees where it flattens out.
(PNG)
