# Supplementary material for: The risk posed by Xanthomonas wilt disease of banana: Mapping of disease hotspots, fronts and vulnerable landscapes
Source: PLoS One. 2019 Apr 2;14(4):e0213691. doi: 10.1371/journal.pone.0213691 (PMC6445462; doi:10.1371/journal.pone.0213691)
Supplement: S1 Appendix — Interactions are indicated with the ‘:’ sign. (PDF) [file pone.0213691.s001.pdf]

S1 Table. Estimates and standard errors of coefficients of the logistic regression model. Interactions are indicated with the ':' sign.

Term	Estimate	Std. Error
(Intercept)	-1.44E+02	6.56E+01
Lon	3.09E-05	1.86E-05
Lat	-7.65E-05	4.79E-05
Alt	6.41E-02	2.03E-02
Prec	-9.08E-02	2.73E-02
Precsq	3.97E-05	1.08E-05
Precmin	1.08E-01	3.13E-01
Precvar	3.31E-01	7.17E-02
Tempsq	2.65E-03	7.75E-04
Market	-6.24E-04	3.51E-04
Vegsq	-5.09E-05	2.16E-05
Man	2.29E-01	1.88E-01
lon:lat	1.40E-11	5.24E-12
lon:alt	-1.18E-08	6.49E-09
lon:tempsq	-4.45E-10	2.47E-10
lon:tempvar	1.10E-08	5.23E-09
lat:alt	5.73E-08	1.10E-08
lat:prec	-1.59E-07	5.08E-08
lat:precsq	6.17E-11	2.03E-11
lat:precmin	-5.28E-07	9.55E-08
lat:precvar	-2.17E-07	1.12E-07
lat:tempsq	2.19E-09	4.14E-10
lat:tempvar	-2.03E-08	6.90E-09
alt:precmin	-2.34E-04	7.15E-05
alt:tempvar	-2.05E-05	4.83E-06
alt:vegsq	3.68E-08	1.47E-08
alt:man	-1.13E-04	4.01E-05
prec:precmin	9.12E-04	3.35E-04
prec:tempvar	3.75E-05	2.51E-05
prec:man	5.82E-04	2.05E-04
precsq:precmin	-3.14E-07	1.28E-07
precsq:tempvar	-1.95E-08	9.80E-09
precsq:man	-2.57E-07	8.13E-08
precmin:tempsq	-9.79E-06	2.70E-06
precmin:man	-8.26E-04	4.45E-04
precvar:tempvar	-1.31E-04	7.73E-05
precvar:man	-4.21E-03	8.43E-04
tempsq:tempvar	-7.82E-07	1.87E-07
tempsq:man	-7.09E-06	1.66E-06

Term	Estimate	Std. Error
man:tempvar	2.49E-04	4.12E-05
