# Supplementary figures and images for: The risk posed by Xanthomonas wilt disease of banana: Mapping of disease hotspots, fronts and vulnerable landscapes
Source: PLoS One. 2019 Apr 2;14(4):e0213691. doi: 10.1371/journal.pone.0213691 (PMC6445462; doi:10.1371/journal.pone.0213691)

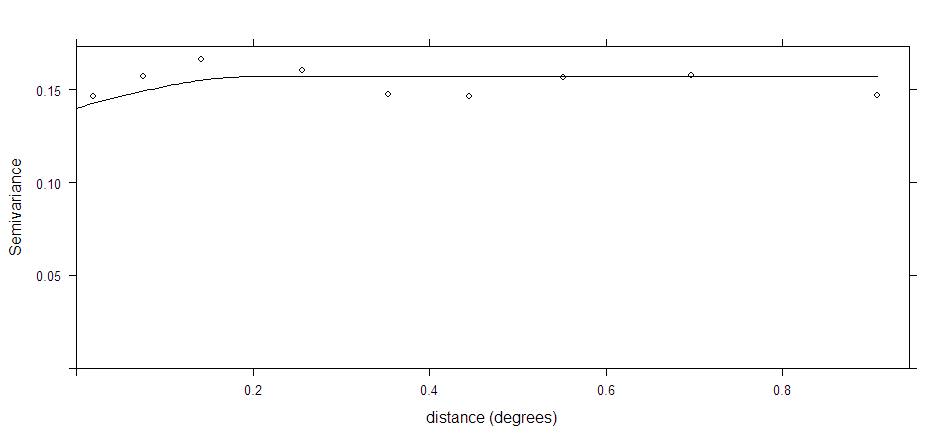

Supplement: S2 Appendix — The line depicts the variogram model, with little spatial autocorrelation until a distance of 0.2 degrees where it flattens out. (PNG) [file pone.0213691.s002.png]
